# Androgen Deprivation Therapy Toxicity and Management for Men Receiving Radiation Therapy

**DOI:** 10.1155/2012/580306

**Published:** 2012-12-30

**Authors:** Matthew E. Johnson, Mark K. Buyyounouski

**Affiliations:** Department of Radiation Oncology, Fox Chase Cancer Center, Philadelphia, PA 19111, USA

## Abstract

Androgen deprivation therapy is commonly used in combination with radiotherapy as part of the definitive treatment for men with clinically localized and locally advanced prostate cancer. Androgen deprivation has been associated with a wide range of iatrogenic effects impacting a variety of body systems including metabolic, musculoskeletal, cardiovascular, neurocognitive, and sexual. This review aims to provide the radiation oncology community with the knowledge to monitor and manage androgen deprivation therapy toxicity in an effort to provide the highest level of care for patients and to minimize the iatrogenic effects of androgen deprivation as much as possible.

## 1. Introduction

As a consequence of the benefits observed in several randomized trials comparing radiotherapy (RT) to RT with androgen deprivation therapy (ADT) [1–10], the use of ADT for the definitive treatment of clinically localized and locally advanced prostate cancer has become more commonplace [11], although recent declines in use have been observed in Canada [12]. With more widespread use of ADT, better awareness of the toxicities associated with ADT is required. This is especially true considering the metabolic effects of ADT and the possible increased cardiovascular risk to patients, highlighted by the Lupron prescribing information that warns “increased risk of heart attack, sudden death, and stroke can occur in men using Lupron Depot” [13]. Radiation oncologists will play an important role in the screening and management of these associated toxicities as advisory statements from the American Heart Association, American Cancer Society, and American Urological Association, endorsed by the American Society for Radiation Oncology, affirms “there is no clear indication for patients for whom ADT is believed to be beneficial to be referred to internists, endocrinologists, or cardiologists for evaluation before initiation of ADT” [14]. While new referrals are not needed, clinicians should be mindful to inform the patient's established physician team including the general practitioner, cardiologist, or endocrinologist regarding the modality and duration of ADT to both request their participation in side effect management and keep them informed as surveillance proceeds. Despite the radiation oncologist's important role, a comprehensive review for the radiation oncologist of ADT toxicity and its management is lacking. The purpose of this paper is to provide the radiation oncology community with the knowledge to monitor and manage ADT toxicity in order to provide the highest quality care for patients and to prevent as much as possible the iatrogenic effects linked to the use of ADT. 

## 2. ADT Administration

Androgen deprivation may be attained through a variety of means when prescribed along with radiation therapy. These have historically included orchiectomy [7], luteinizing hormone-releasing hormone (LHRH) agonists [2, 6], antiandrogens [15], or combination of an anti-androgen and LHRH agonist [4, 10, 16, 17].

## 3. Metabolic Effects

The use of ADT has been associated with a wide range of metabolic alterations including weight gain, changes in lipid profile and worsening insulin resistance [18]. The impact and management of these alterations and their impact on cardiovascular morbidity and mortality are discussed here.

### 3.1. Weight Gain

Men on ADT often note an increase in body fat and redistribution of weight. As few as 48 weeks of ADT can increase BMI by 2.4% [19]. Small prospective studies have demonstrated an 11% increase in fat mass, 16.5% increase in total abdominal fat, and a 3.8% decrease in lean body mass with one year of ADT [20, 21]. The impact of weight gain alone may be significant: a large European prospective study has shown that increasing waist circumference and BMI have been associated with higher risk of death in the general population [22]. Men on ADT should be made aware of the possibility of weight gain so that they can monitor their weight and adjust their diet and activity level as needed during the course of therapy. The American Heart Association suggests at least 150 minutes per week of moderate exercise or 75 minutes per week of vigorous exercise. An example beginner exercise regimen may be walking 30 minutes a day, five days a week. The US Department of Health and Human Services and the US Department of Agriculture have jointly published the Dietary Guidelines every five years since 1980 [23]. Appropriate caloric intake with a diet high in fruits and vegetables while low in solid fats, sugars, and salts, is key to a healthy diet. Patients can estimate their dietary needs and learn more at ChooseMyPlate.gov.

### 3.2. Lipids

The effect of ADT on the lipid profile can also be significant. Less than one year of ADT can increase total cholesterol by 9%, increase LDL by 7.3%, and increase triglycerides by 26.5% [19]. The importance of lipid management has been seen in a meta-analysis of 900,000 people in the general population, where total cholesterol level has been directly associated with cardiovascular mortality at all blood pressure levels [24]. One management option for ADT-induced dyslipidemia is toremifene, which has been shown to significantly decrease total cholesterol, LDL, and triglyceride levels, as well as increase HDL levels in a phase III randomized trial [25]. The National Cholesterol Education Program Adult Treatment Panel III (NCEP ATP III) guidelines define the standard of care for lipid management in the general population and, as there are no guidelines specific to the population on ADT, these guidelines should generally be used to direct the management of ADT-induced dyslipidemia. Diet and lifestyle interventions, such as those discussed above, remain the first-line intervention, but statins should be initiated if needed to attain goals [26]. 

### 3.3. Insulin Resistance

Insulin resistance is associated with the use of ADT and can lead to an increase in the diagnosis of diabetes mellitus. Twelve weeks of ADT has been demonstrated to increase median serum insulin levels from 11.8 to 19.3 mU/L and reduce insulin sensitivity by 12.9% [27, 28]. An observational study of 37,000 men receiving ADT for prostate cancer noted an aHR of 1.28 for incident cases of diabetes mellitus [29]. This increased risk suggests a benefit to screening for diabetes in this population. The American Diabetes Association recommends screening at-risk populations with either a fasting plasma glucose test, hemoglobin A1c, or 2-hour oral glucose tolerance test. A baseline screening may help identify men with preexisting insulin resistance that may be at higher risk of diabetes during the course of ADT [30]. The most commonly used interventions for those with insulin resistance include lifestyle interventions and metformin. A randomized trial of 3,200 people with elevated glucose concentrations was randomized to lifestyle intervention, metformin (850 mg BID), or placebo. Lifestyle intervention reduced diabetes incidence by 58%, significantly more than metformin, which reduced it by 31% [31]. Once again, lifestyle intervention should be recommended to all patients receiving ADT, including education on diet, exercise, and weight loss.

## 4. Cardiovascular

 The metabolic changes seen in men receiving ADT are concerning for their possible contribution to cardiovascular morbidity and mortality. Cardiovascular disease is already the leading cause of mortality in men with early stage prostate cancer [32], so any possible increase in this risk should be taken seriously. However, the existing literature on the relationship between ADT and cardiovascular morbidity is somewhat mixed. A large VA observational study demonstrated an aHR of 1.28 for myocardial infarction and 1.22 for sudden cardiac death for men on ADT [29], while another large retrospective study demonstrated a 20% increase in cardiovascular events with 12 months of ADT [14]. This increase has also been shown in men older than 65, who were noted to have a shorter time to fatal MI with a history of ADT use for as short as 3 months [33]. Other analyses have shown no correlation between ADT use and cardiovascular mortality. For example, the 8-year followup of RTOG 85-31 [34] and 10-year followup of EORTC 22863 [35] conclude that GnRH agonist use does not significantly increase cardiovascular mortality. Recently, a meta-analysis of over 4,000 patients was unable to show an increased risk of cardiovascular death regardless of ADT duration [36]. Nonetheless, the metabolic changes seen during the use of ADT have been demonstrated to confer excess cardiovascular risk in the general population, and close monitoring of modifiable cardiovascular risk factors is warranted. The science advisory statement from the American Heart Association, American Cancer Society, and American Urological Association recommends annual monitoring of blood pressure, lipid profile, and glucose level for men receiving ADT [14]. 

While lipid levels and glucose tolerance are adversely affected by ADT as described in the metabolic section, blood pressure is not known to be affected by ADT. Blood pressure, however, is a modifiable cardiovascular risk factor and should be tightly monitored during ADT use to minimize overall cardiovascular risk. Blood pressure should be managed similar to that recommended for the general population: prehypertension should be aggressively treated with lifestyle modification, while stage 1 hypertension should be managed with lifestyle modification and antihypertensive medication. Stage 2 hypertension is treated similarly to stage I with the addition of a diuretic [37]. 

## 5. Musculoskeletal

### 5.1. Muscle Loss

Patients receiving ADT often report loss of muscle mass and muscle weakness, and ADT has been demonstrated to be associated with a decrease in muscle strength and functional performance [38]. Lean body mass has also been shown to decrease by 3.8% with one year of ADT [21]. Two randomized controlled trials have reported on the utility of a structured exercise program to counteract this loss of muscle mass. One trial randomized patients to 12-weeks of a resistance exercise program, done 3 times per week [39]. A second trial reported on a 12 week program of twice per week combined aerobic and resistance exercise [40]. Aerobic exercise included 15–20 minutes of cycling, walking, or jogging at 65–80% maximum exertion. Resistance exercises included chest press, seated row, shoulder press, triceps extension, leg press, leg extension, leg curl, abdominal crunches, and flexibility exercises. Both trials found that participants in the exercise programs had improved muscle strength, muscle mass, quality of life, and reduced fatigue. At the time of the initiation of ADT, patients should be provided recommendations regarding an exercise program that can be used to maintain muscular fitness during the course of ADT. 

### 5.2. Osteoporosis

Much has been published regarding the link between osteoporosis and ADT and subsequent fracture risk following therapy. ADT is used in a population where the baseline prevalence of osteopenia is as high as 46% and osteoporosis is 14% even prior to the initiation of any treatment [41]. Mechanistically, ADT has been shown to lead to microarchitectural decay in bone after 12 months of treatment [42]. An 8.5% decrease in bone mineral density (BMD) can be seen even after 48 weeks of ADT [43]. This has been linked to an increased risk for osteoporotic fracture during and after the use of ADT. A retrospective study of over 12,000 men demonstrated a relative risk of 1.21 for any fracture, 1.18 for vertebral fracture, and 1.76 for hip fracture for patients who had received ADT [44]. This increased risk has also been correlated to a number of doses of ADT administered [45]. Long-term ADT can increase the rate of osteoporosis to as high as 81% after 10 years of treatment [46].

Supplementation with 1200 mg calcium and 800 IU vitamin D daily has been shown to reduce the incidence of osteoporotic fractures in the general population over 50 years old [47], but calcium and vitamin D alone are not sufficient to prevent bone loss in men undergoing ADT [43]. The addition of a bisphosphonate—pamindronate [43, 48], alendronate [49], or zoledronic acid [50, 51]—has been shown to maintain or increase BMD during ADT and is generally well tolerated. Other agents have shown to be effective as well. A trial of 1,468 men receiving ADT randomized to denosumab, a RANKL inhibitor, given 60 mg SQ every 6 months versus placebo demonstrated a 5.6% gain in BMD at 2 years, versus a 1% loss in the placebo group, and was found to decrease the risk of vertebral fractures at 36 months to 1.5% from 3.9% in the placebo group [52]. Denosumab is currently FDA approved for both the prevention of ADT-induced bone loss and for the prevention of skeletal related events in patients with metastatic cancer, but is associated with severe hypocalcemia and osteonecrosis of the jaw [53, 54]. The selective estrogen receptor modulators raloxifene [55] and toremifene have also been used to improve BMD. A phase III randomized controlled trial of toremifene in 646 men demonstrated a reduction in new vertebral fractures from 4.9% in placebo group to 2.5% [56]. However, concern exists with hormonal agents for both their increased risk of venous thromboembolism and their modulation of hormone cascades, which has the potential to diminish the effectiveness of ADT.

Bone density can be assessed with dual-energy X-ray absorptiometry (DEXA). DEXA may be used at the start of ADT and monitored subsequently every 1-2 years as indicated. The National Osteoporosis Foundation recommends supplemental calcium (1,200 mg daily) and vitamin D3 (800–1,000 IU daily) for all men over age 50 years and an additional treatment for men when the 10-year probability of hip fracture is ≥3% or the 10-year probability of a major osteoporosisrelated fracture is ≥20% [57]. Encouragement of lifestyle measures (smoking cessation, moderating alcohol intake, and increasing weight-bearing exercise), along with calcium and vitamin D supplementation, should be routinely performed. Bisphosphonate use should be considered, especially for those men with osteoporosis or osteopenia at baseline [18, 58] or 10-year osteoporotic fracture risk of >20% by the FRAX model [59].

### 5.3. Hot Flushes

Hot flushes, also known as hot flashes or vasomotor flushing, are a common side effect of ADT, occurring in 80% of patients undergoing treatment. Up to 27% of patients receiving ADT report hot flushes to be the most troublesome treatment-related side effect [60]. Hot flushes are described as unpredictable episodes of intense warmth, most commonly occurring in the face and upper body that is often accompanied by diaphoresis that usually last less than 5 minutes [61]. Natural and complementary approaches to hot flushes have been tested with varying success. Herbal supplements, such as black cohosh, ginseng, licorice, and turmeric may have some benefit, but have not shown efficacy in randomized controlled trials. Weekly acupuncture for 12 weeks was able to demonstrate a 78% decrease in a hot flush symptom score and should be considered a reasonable treatment strategy [62]. Medical therapy with antidepressants and hormonal agents have also been studied. A small series examined transdermal estrogen, with 83% of men reporting an improvement in hot flushes, although an increase in breast swelling or nipple tenderness was reported [63]. Megestrol acetate given at 40 mg per day has also been shown to be effective at reducing the frequency of hot flushes [64]. A randomized controlled trial of patients having >14 hot flushes per week after 6 months of ADT was randomized to venlafaxine 75 mg daily, medroxyprogesterone acetate 20 mg daily, or cyproterone 100 mg daily. All three agents were able to decrease the frequency of hot flushes although both hormonal agents were more effective than venlafaxine [60]. However, there is a concern that hormonal agents may interact with the ADT and may even cause an increase in prostate specific antigen (PSA). As a result, venlafaxine, which is generally well tolerated, is more often considered the first-line treatment for ADT-induced hot flushes [65].

## 6. Neurocognitive

### 6.1. Memory

There has been some concern for the decline of memory and neurocognitive function during ADT although existing data on this topic is highly conflicted. One prospective Australian study utilized interval neurocognitive battery testing in 50 men treated with ADT and found that 48% demonstrated decline in one cognitive task and 14% in two or more tasks at one year [66], while a prospective trial of 244 patients was unable to demonstrate any evidence of neurocognitive function decline after ADT use for 12 months on a 12-test battery [67]. 

### 6.2. Depression

Depressive disorders have been reported to be more common in men receiving ADT. The proportion of men developing at least one depressive, cognitive, or constitutional disorder was 31.3% in men with prostate cancer undergoing ADT, compared to 23.7% in men with prostate cancer not receiving ADT, and 22.9% in a noncancer control group [68]. Men getting ADT should be informed of this potential side effect so that they can have increased awareness and seek rapid intervention. Radiation oncologists seeing men on ADT should be cognizant of mood disorders so that affected men can be appropriately referred for treatment.

## 7. Sexual

### 7.1. Erectile Dysfunction, Loss of Libido

Onset of loss of libido is frequently seen within the first few months of initiation of ADT and is often followed by erectile dysfunction. Up to 73% of men ceased engaging in sexual activity after initiation of treatment, and 38% of patients getting an LHRH agonist reported sexual function as a “moderate” or “big” problem [69]. As in the general population, first-line treatment for ADT induced erectile dysfunction is phosphodiesterase-5 inhibitors, although these agents have been shown to have a reduced response rate in men who have received ADT. 4 months of ADT reduce the response rate to sildenafil at 24 months from 61% to 47% [70]. Other options for the management of erectile dysfunction follow the same paradigm as that for the general population and include penile implants, vacuum devices, and intracavernosal injections [65].

### 7.2. Decreased Penile and Testicle Size, Thinning of Body Hair

Men receiving ADT often notice a decrease in both penile and testicle size, as well as a thinning of body hair. For some men, these findings have a significant impact on self-image and quality of life. There are currently no interventions to reverse these side effects, so pretreatment counseling on expected side effects remains important.

### 7.3. Gynecomastia and Breast Pain

ADT may lead to the development of gynecomastia and breast pain in as many as 70% of patients [71]. This can also have a significant effect on a patient's sense of self-image and their overall quality of life. Two approaches have been taken in the prophylactic setting: tamoxifen 10 mg daily for 24 weeks and breast irradiation, both of which have been shown to significantly decrease the incidence of gynecomastia [71, 72]. Radiotherapy may include either single fraction (9–12 Gy) or fractionated treatment (12–15 Gy given in 2-3 fractions) [73–75]. Once gynecomastia and breast pain have developed, the same two approaches have been used for treatment, but results in this setting favor the use of tamoxifen given 10–20 mg per day for 12 weeks [71, 72]. Although not studied, concern has been raised about the possibility of a synergistic neurocognitive impact of tamoxifen and ADT together. The aromatase inhibitor anastrozole has also been compared to tamoxifen for the treatment of gynecomastia and breast pain, with anastrozole having no significant reduction in symptoms and tamoxifen reducing gynecomastia from 73% to10% and breast pain from 39% to 6% [76]. 

## 8. Conclusion

The administration of ADT is associated with a diverse set of known side effects which, when compiled, have a significant impact on a prostate cancer patient's quality of life, overall health, and possibly mortality. While the impact of these side effects can be diminished by early diagnosis and treatment, many of the current management strategies discussed in this paper do not yet appear in consensus guidelines for the treatment of prostate cancer. A compilation of management suggestions can be found in Table 1. Radiation oncologists will serve an important role in advocating for the screening, diagnosis, and management of these side effects, as much of the current role for ADT is its concurrent use in combination with radiation therapy. Improved awareness of the far-reaching effects of ADT by radiation oncologists will lead to a better identification of these ADT side effects and improved multidisciplinary care and, as a result, will mitigate much of the short-term and long-term impact that they can have on the patient.

## Figures and Tables

**Table 1:** Suggested management for patients receiving androgen deprivation therapy.

At time of ADT^1^ initiation	

Patient education on spectrum of expected side effects	
Education on initiation of exercise program	
Patient resources at http://go4life.nia.nih.gov/	
Education on diet and weight management	
Obtain baseline weight	
Patient resources at http://www.choosemyplate.gov/	
Inform primary care physician of modality and duration of ADT	
Baseline lipid panel	
Blood pressure measurement	
Insulin resistance screening	
Smoking cessation	
Baseline DEXA^2^ scan	
Assess fracture risk at http://www.shef.ac.uk/FRAX/	

Year 1	

Ensure continued followup with primary care physician	
Blood pressure management	
Lipid monitoring	
Weight management	
Diabetes screening	
Ongoing screening for depression, sexual side effects, and hot flushes	

Year 2-3	

Ensure continued followup with primary care physician	
Blood pressure management	
Lipid monitoring	
Weight management	
Diabetes screening	
Ongoing screening for depression, sexual side effects, and hot flushes	
DEXA scan	
Reassess fracture risk at http://www.shef.ac.uk/FRAX/	

^1^Androgen deprivation therapy, ^2^dual-energy X-ray absorptiometry.

## References

[1] Pilepich MV, Winter K, John MJ (2001). Phase III radiation therapy oncology group (RTOG) trial 86-10 of androgen deprivation adjuvant to definitive radiotherapy in locally advanced carcinoma of the prostate. *International Journal of Radiation Oncology Biology Physics*.

[2] Bolla M, Collette L, Blank L (2002). Long-term results with immediate androgen suppression and external irradiation in patients with locally advanced prostate cancer (an EORTC study): a phase III randomised trial. *The Lancet*.

[3] Hanks GE, Pajak TF, Porter A (2003). Phase III trial of long-term adjuvant androgen deprivation after neoadjuvant hormonal cytoreduction and radiotherapy in locally advanced carcinoma of the prostate: the radiation therapy oncology group protocol 92-02. *Journal of Clinical Oncology*.

[4] D’Amico AV, Manola J, Loffredo M, Renshaw AA, DellaCroce A, Kantoff PW (2004). 6-Month androgen suppression plus radiation therapy vs radiation therapy alone for patients with clinically localized prostate cancer: a randomized controlled trial. *The Journal of the American Medical Association*.

[5] Laverdière J, Nabid A, de Bedoya LD (2004). The efficacy and sequencing of a short course of androgen suppression on freedom from biochemical failure when administered with radiation therapy for T2-T3 prostate cancer. *Journal of Urology*.

[6] Pilepich MV, Winter K, Lawton CA (2005). Androgen suppression adjuvant to definitive radiotherapy in prostate carcinoma—long-term results of phase III RTOG 85-31. *International Journal of Radiation Oncology Biology Physics*.

[7] Granfors T, Modig H, Damber JE, Tomic R (2006). Long-term followup of a randomized study of locally advanced prostate cancer treated with combined orchiectomy and external radiotherapy versus radiotherapy alone. *Journal of Urology*.

[8] Bolla M, de Reijke TM, van Tienhoven G (2009). Duration of androgen suppression in the treatment of prostate cancer. *The New England Journal of Medicine*.

[9] Denham JW, Steigler A, Lamb DS (2011). Short-term neoadjuvant androgen deprivation and radiotherapy for locally advanced prostate cancer: 10-year data from the TROG 96.01 randomised trial. *The Lancet Oncology*.

[10] Jones CU, Hunt D, McGowan DG (2011). Radiotherapy and short-term androgen deprivation for localized prostate cancer. *The New England Journal of Medicine*.

[11] Shahinian VB, Kuo YF, Freeman JL, Orihuela E, Goodwin JS (2005). Increasing use of gonadotropin-releasing hormone agonists for the treatment of localized prostate carcinoma. *Cancer*.

[12] Krahn M, Bremner KE, Tomlinson G, Luo J, Ritvo P, Naglie G (2011). Androgen deprivation therapy in prostate cancer: are rising concerns leading to falling use?. *British Journal of Urology International*.

[13] Lupron depot prescribing information. http://www.lupron.com/prescribing-information.cfm.

[14] Levine GN, D’Amico AV, Berger P (2010). Androgen-deprivation therapy in prostate cancer and cardiovascular risk: a science advisory from the American heart association, American cancer society, and American urological association: endorsed by the American society for radiation oncology. *CA: Cancer Journal for Clinicians*.

[15] Shipley W (2011). Initial report of RTOG, 9601, a phase III trial in prostate cancer: effect of anti-androgen therapy (AAT) with bicalutamide during and after radiation therapy (RT) on freedom from progression and incidence of metastatic disease in patients following radical prostatectomy (RP) with pT2-3, N0 disease and elevated PSA levels. *Journal of Clinical Oncology*.

[16] Roach M, Bae K, Speight J (2008). Short-term neoadjuvant androgen deprivation therapy and external-beam radiotherapy for locally advanced prostate cancer: long-term results of RTOG 8610. *Journal of Clinical Oncology*.

[17] Widmark A, Klepp O, Solberg A (2009). Endocrine treatment, with or without radiotherapy, in locally advanced prostate cancer (SPCG-7/SFUO-3): an open randomised phase III trial. *The Lancet*.

[18] Grossmann M, Hamilton EJ, Gilfillan C, Bolton D, Joon DL, Zajac JD (2011). Bone and metabolic health in patients with non-metastatic prostate cancer who are receiving androgen deprivation therapy. *Medical Journal of Australia*.

[19] Smith MR, Finkelstein JS, McGovern FJ (2002). Changes in body composition during androgen deprivation therapy for prostate cancer. *Journal of Clinical Endocrinology and Metabolism*.

[20] Smith MR, Lee H, McGovem F (2008). Metabolic changes during gonadotropin-releasing hormone agonist therapy for prostate cancer: differences from the classic metabolic syndrome. *Cancer*.

[21] Smith MR (2004). Changes in fat and lean body mass during androgen-deprivation therapy for prostate cancer. *Urology*.

[22] Pischon T, Boeing H, Hoffmann K (2008). General and abdominal adiposity and risk of death in Europe. *The New England Journal of Medicine*.

[23] U.S. Department of Agriculture (2010). *Dietary Guidelines for Americans, 2010*.

[24] Prospective Studies Collaboration (2007). Blood cholesterol and vascular mortality by age, sex, and blood pressure: a meta-analysis of individual data from 61 prospective studies with 55,000 vascular deaths. *The Lancet*.

[25] Smith MR, Malkowicz SB, Chu F (2008). Toremifene improves lipid profiles in men receiving androgen-deprivation therapy for prostate cancer: interim analysis of a multicenter phase III study. *Journal of Clinical Oncology*.

[26] National Cholesterol Education Program (2002). Third report of the national cholesterol education program (NCEP) expert panel on detection, evaluation, and treatment of high blood cholesterol in adults (adult treatment panel III) final report. *Circulation*.

[27] Smith MR, Lee H, Fallon MA, Nathan DM (2008). Adipocytokines, obesity, and insulin resistance during combined androgen blockade for prostate cancer. *Urology*.

[28] Smith JC, Bennett S, Evans LM (2001). The effects of induced hypogonadism on arterial stiffness, body composition, and metabolic parameters in males with prostate cancer. *Journal of Clinical Endocrinology and Metabolism*.

[29] Keating NL, O’Malley AJ, Freedland SJ, Smith MR (2010). Diabetes and cardiovascular disease during androgen deprivation therapy: observational study of veterans with prostate cancer. *Journal of the National Cancer Institute*.

[30] American Diabetes Association (2012). Standards of medical care in diabetes—2012. *Diabetes Care*.

[31] Knowler WC, Barrett-Connor E, Fowler SE (2002). Reduction in the incidence of type 2 diabetes with lifestyle intervention or metformin. *The New England Journal of Medicine*.

[32] Grossmann M, Zajac JD (2011). Androgen deprivation therapy in men with prostate cancer: how should the side effects be monitored and treated?. *Clinical Endocrinology*.

[33] D’Amico AV, Denham JW, Crook J (2007). Influence of androgen suppression therapy for prostate cancer on the frequency and timing of fatal myocardial infarctions. *Journal of Clinical Oncology*.

[34] Efstathiou JA, Bae K, Shipley WU (2009). Cardiovascular mortality after androgen deprivation therapy for locally advanced prostate cancer: Rtog 85-31. *Journal of Clinical Oncology*.

[35] Bolla M, van Tienhoven G, Warde P (2010). External irradiation with or without long-term androgen suppression for prostate cancer with high metastatic risk: 10-year results of an EORTC randomised study. *The Lancet Oncology*.

[36] Nguyen PL, Je Y, Schutz FAB (2011). Association of androgen deprivation therapy with cardiovascular death in patients with prostate cancer: a meta-analysis of randomized trials. *The Journal of the American Medical Association*.

[37] Chobanian AV, Bakris GL, Black HR (2003). The seventh report of the joint national committee on prevention, detection, evaluation, and treatment of high blood pressure: the JNC 7 report. *The Journal of the American Medical Association*.

[38] Galvão DA, Taaffe DR, Spry N, Joseph D, Turner D, Newton RU (2009). Reduced muscle strength and functional performance in men with prostate cancer undergoing androgen suppression: a comprehensive cross-sectional investigation. *Prostate Cancer and Prostatic Diseases*.

[39] Segal RJ, Reid RD, Courneya KS (2003). Resistance exercise in men receiving androgen deprivation therapy for prostate cancer. *Journal of Clinical Oncology*.

[40] Galvão DA, Taaffe DR, Spry N, Joseph D, Newton RU (2010). Combined resistance and aerobic exercise program reverses muscle loss in men undergoing androgen suppression therapy for prostate cancer without bone metastases: a randomized controlled trial. *Journal of Clinical Oncology*.

[41] Berruti A, Dogliotti L, Terrone C (2002). Changes in bone mineral density, lean body mass and fat content as measured by dual energy x-ray absorptiometry in patients with prostate cancer without apparent bone metastases given androgen deprivation therapy. *Journal of Urology*.

[42] Hamilton EJ, Ghasem-Zadeh A, Gianatti E (2010). Structural decay of bone microarchitecture in men with prostate cancer treated with androgen deprivation therapy. *Journal of Clinical Endocrinology and Metabolism*.

[43] Smith MR, McGovern FJ, Zietman AL (2001). Pamidronate to prevent bone loss during androgen-deprivation therapy for prostate cancer. *The New England Journal of Medicine*.

[44] Smith MR, Boyce SP, Moyneur E, Duh MS, Raut MK, Brandman J (2006). Risk of clinical fractures after gonadotropin-releasing hormone agonist therapy for prostate cancer. *Journal of Urology*.

[45] Shahinian VB, Kuo YF, Freeman JL, Goodwin JS (2005). Risk of fracture after androgen deprivation for prostate cancer. *The New England Journal of Medicine*.

[46] Morote J, Morin JP, Orsola A (2007). Prevalence of osteoporosis during long-term androgen deprivation therapy in patients with prostate cancer. *Urology*.

[47] Ebeling PR (2008). Osteoporosis in men. *The New England Journal of Medicine*.

[48] Diamond TH, Winters J, Smith A (2001). The antiosteoporotic efficacy of intravenous pamidronate in men with prostate carcinoma receiving combined androgen blockade: a double blind, randomized, placebo-controlled crossover study. *Cancer*.

[49] Greenspan SL, Nelson JB, Trump DL, Resnick NM (2007). Effect of once-weekly oral alendronate on bone loss in men receiving androgen deprivation therapy for prostate cancer: a randomized trial. *Annals of Internal Medicine*.

[50] Michaelson MD, Kaufman DS, Lee H (2007). Randomized controlled trial of annual zoledronic acid to prevent gonadotropin-releasing hormone agonist-induced bone loss in men with prostate cancer. *Journal of Clinical Oncology*.

[51] Smith MR, Eastham J, Gleason DM, Shasha D, Tchekmedyian S, Zinner N (2003). Randomized controlled trial of zoledronic acid to prevent bone loss in men receiving androgen deprivation therapy for nonmetastatic prostate cancer. *Journal of Urology*.

[52] Smith MR, Egerdie B, Toriz NH (2009). Denosumab in men receiving androgen-deprivation therapy for prostate cancer. *The New England Journal of Medicine*.

[53] Prolia prescribing information. http://pi.amgen.com/united_states/prolia/prolia_pi.pdf.

[54] Xgeva prescribing information. http://pi.amgen.com/united_states/xgeva/xgeva_pi.pdf.

[55] Smith MR, Fallon MA, Lee H, Finkelstein JS (2004). Raloxifene to prevent gonadotropin-releasing hormone agonist-induced bone loss in men with prostate cancer: a randomized controlled trial. *Journal of Clinical Endocrinology and Metabolism*.

[56] Smith MR, Morton RA, Barnette KG (2010). Toremifene to reduce fracture risk in men receiving androgen deprivation therapy for prostate cancer. *Journal of Urology*.

[57] National Osteoporosis Foundation (2010). *Clinician's Guide to Prevention and Treatment of Osteoporosis*.

[58] Saad F, Adachi JD, Brown JP (2008). Cancer treatment-induced bone loss in breast and prostate cancer. *Journal of Clinical Oncology*.

[59] Watts NB, Lewiecki EM, Miller PD, Baim S (2008). National osteoporosis foundation 2008 clinician’s guide to prevention and treatment of osteoporosis and the world health organization fracture risk assessment tool (FRAX): what they mean to the bone densitometrist and bone technologist. *Journal of Clinical Densitometry*.

[60] Irani J, Salomon L, Oba R, Bouchard P, Mottet N (2010). Efficacy of venlafaxine, medroxyprogesterone acetate, and cyproterone acetate for the treatment of vasomotor hot flushes in men taking gonadotropin-releasing hormone analogues for prostate cancer: a double-blind, randomised trial. *The Lancet Oncology*.

[61] Michaelson MD, Cotter SE, Gargollo PC, Zietman AL, Dahl DM, Smith MR (2008). Management of complications of prostate cancer treatment. *CA: Cancer Journal for Clinicians*.

[62] Frisk J, Spetz AC, Hjertberg H, Petersson B, Hammar M (2009). Two modes of acupuncture as a treatment for hot flushes in men with prostate cancer—a prospective multicenter study with long-term follow-up. *European Urology*.

[63] Gerber GS, Zagaja GP, Ray PS, Rukstalis DB (2000). Transdermal estrogen in the treatment of hot flushes in men with prostate cancer. *Urology*.

[64] Loprinzi CL, Michalak JC, Quella SK (1994). Megestrol acetate for the prevention of hot flashes. *The New England Journal of Medicine*.

[65] Sharifi N, Gulley JL, Dahut WL (2005). Androgen deprivation therapy for prostate cancer. *The Journal of the American Medical Association*.

[66] Salminen E, Portin R, Korpela J (2003). Androgen deprivation and cognition in prostate cancer. *British Journal of Cancer*.

[67] Alibhai S (2010). Impact of 12 months of androgen deprivation therapy (ADT) on cognitive function in men with nonmetaastatic prostate cancer (PC): a matched cohort study. *Journal of Clinical Oncology*.

[68] Shahinian VB, Kuo YF, Freeman JL, Goodwin JS (2006). Risk of the “androgen deprivation syndrome” in men receiving androgen deprivation for prostate cancer. *Archives of Internal Medicine*.

[69] Potosky AL, Knopf K, Clegg LX (2001). Quality-of-life outcomes after primary androgen deprivation therapy: results from the prostate cancer outcomes study. *Journal of Clinical Oncology*.

[70] Teloken PE, Ohebshalom M, Mohideen N, Mulhall JP (2007). Analysis of the impact of androgen deprivation therapy on sildenafil citrate response following radiation therapy for prostate cancer. *Journal of Urology*.

[71] Di Lorenzo G, Perdoná S, de Placido S (2005). Gynecomastia and breast pain induced by adjuvant therapy with bicalutamide after radical prostatectomy in patients with prostate cancer: the role of tamoxifen and radiotherapy. *Journal of Urology*.

[72] Perdonà S, Autorino R, de Placido S (2005). Efficacy of tamoxifen and radiotherapy for prevention and treatment of gynaecomastia and breast pain caused by bicalutamide in prostate cancer: a randomised controlled trial. *The Lancet Oncology*.

[73] Cook S, Rodríguez-Antunez A (1973). Pre-estrogen irradiation of the breast to prevent gynecomastia. *The American Journal of Roentgenology, Radium Therapy, and Nuclear Medicine*.

[74] Fass D, Steinfeld A, Brown J, Tessler A (1986). Radiotherapeutic prophylaxis of estrogen-induced gynecomastia: a study of late sequela. *International Journal of Radiation Oncology Biology Physics*.

[75] Gunderson L, Tepper J (2011). *Clinical Radiation Oncology*.

[76] Boccardo F, Rubagotti A, Battaglia M (2005). Evaluation of tamoxifen and anastrozole in the prevention of gynecomastia and breast pain induced by bicalutamide monotherapy of prostate cancer. *Journal of Clinical Oncology*.

